# Ciprofloxacin Affects Host Cells by Suppressing Expression of the Endogenous Antimicrobial Peptides Cathelicidins and Beta-Defensin-3 in Colon Epithelia

**DOI:** 10.3390/antibiotics3030353

**Published:** 2014-07-25

**Authors:** Protim Sarker, Akhirunnesa Mily, Abdullah Al Mamun, Shah Jalal, Peter Bergman, Rubhana Raqib, Gudmundur H. Gudmundsson, Birgitta Agerberth

**Affiliations:** 1 Centre for Vaccine Science, International Centre for Diarrhoeal Disease Research, Bangladesh (icddr,b), 68 Shaheed Tajuddin Ahmed Sharani, Mohakhali, Dhaka 1212, Bangladesh; E-Mails: protim@icddrb.org (P.S.); mily@icddrb.org (A.M.); mamun104@gmail.com (A.A.M.); rubhana@icddrb.org (R.R.); 2 Department of Laboratory Medicine, Division of Clinical Microbiology (F68), Karolinska University Hospital Huddinge, S-141 86 Stockholm, Sweden; E-Mails: Shah.Jalal@ki.se (S.J.); Peter.Bergman@ki.se (P.B.); 3 Institute of Biology and Biomedical Center, University of Iceland, 101 Reykjavik, Iceland; E-Mail: ghrafn@hi.is

**Keywords:** antibiobic, microbiota, butyrate, histone modifications, host defense peptides, LL-37, innate immunity, impaired immune responses, *Clostridium difficile*, antibiotic-associated diarrhea

## Abstract

Antibiotics exert several effects on host cells including regulation of immune components. Antimicrobial peptides (AMPs), e.g., cathelicidins and defensins display multiple functions in innate immunity. In colonic mucosa, cathelicidins are induced by butyrate, a bacterial fermentation product. Here, we investigated the effect of antibiotics on butyrate-induced expression of cathelicidins and beta-defensins in colon epithelial cells. Real-time PCR analysis revealed that ciprofloxacin and clindamycin reduce butyrate-induced transcription of the human cathelicidin LL-37 in the colonic epithelial cell line HT-29. Suppression of LL-37 peptide/protein by ciprofloxacin was confirmed by Western blot analysis. Immunohistochemical analysis demonstrated that ciprofloxacin suppresses the rabbit cathelicidin CAP-18 in rectal epithelia of healthy and butyrate-treated *Shigella*-infected rabbits. Ciprofloxacin also down-regulated butyrate-induced transcription of the human beta-defensin-3 in HT-29 cells. Microarray analysis of HT-29 cells revealed upregulation by butyrate with subsequent down-regulation by ciprofloxacin of additional genes encoding immune factors. Dephosphorylation of histone H3, an epigenetic event provided a possible mechanism of the suppressive effect of ciprofloxacin. Furthermore, LL-37 peptide inhibited *Clostridium difficile* growth *in vitro*. In conclusion, ciprofloxacin and clindamycin exert immunomodulatory function by down-regulating AMPs and other immune components in colonic epithelial cells. Suppression of AMPs may contribute to the overgrowth of *C. difficile*, causing antibiotic-associated diarrhea.

## 1. Introduction

Antimicrobial peptides/proteins (AMPs) are important effectors of the immediate host defense, exerting antimicrobial activity and immunomodulation [1,2,3,4]. Defensins and cathelicidins are the two major classes of AMPs in mammals. LL-37 is the sole cathelicidin peptide in human and its orthologs in rabbit, mouse, and rat are CAP-18, mCRAMP and rCRAMP, respectively [5]. LL-37 and its orthologs are cationic, amphipathic, α-helical peptides. Defensins are cationic peptides, having a characteristic anti-parallel β-sheet fold and consist of six conserved cysteine residues forming three disulphide bonds [6,7]. Based on the size and disulfide linkage, defensins are classified into α-, β-, and θ-defensins. In human, six α-defensins, e.g., human neutrophil peptides (HNP)-1 to -4, human defensins (HD)-5 and -6, and four β-defensins, e.g., human β-defensin (HBD)-1 to -4 have, thus far, been characterized [6,8]. LL-37 and/or HBDs have been implicated in several functions including killing of microorganisms, neutralization of lipopolysaccharide, immune regulation, regulation of normal flora, wound healing, angiogenesis, and anticancer activities [3,5,6,8,9,10,11,12,13,14].

LL-37 is expressed in neutophils, monocytes, lymphocytes, mast cells eosinophils, dendritic cells, and epithelial cells of different organs [5,14]. HBDs are predominantly expressed in epithelial cells [8]. The expression of LL-37 and HBDs can be modulated by different stimuli in a cell and tissue specific manner [15]. Butyrate, a bacterial fermentation product in colon, upregulates cathelicidins in colonic epithelial cells of human and rabbit [16,17], and, thus, playing an important role in host-microbes interaction in the colonic mucosa.

Antibiotics, apart from exerting bactericidal/bacteriostatic effects on pathogens, can render pathogens susceptible to the host immune system, such as killing of bacteria by polymorphonuclear neutrophils (PMNs) [18]. On the other hand, by inducing production and release of microbial components, antibiotics may provoke proinflammatory responses in host cells [19]. Numerous antibiotics also have direct modulatory effects on immune functions [20]. Moreover, antibiotic treatment disrupts the normal colonic flora that may allow colonization and secondary infections by enteropathogens such as *Clostridium difficile*, *Clostridium perfringens*, *Staphylococcus aureus*, and *Salmonella* spp. [21]. Alteration of the microbiota also affects immune homeostasis including expression of AMPs, leading to infections such as increasing susceptibility to *Listeria monocytogenes* [22]. *C. difficile* is the major cause of antibiotic associated diarrhoea (AAD), accounting for 10%–20% of all AAD cases [21]. Clindamycin, extended-spectrum penicillin, cephalosporin and fluoroquinolones including ciprofloxacin are the major antibiotics implicated in *C. difficile* associated diarrhoea (CDAD) [23].

In this study, we determined the effect of several antibiotics on the constitutive and butyrate-induced expression of cathelicidins in colon epithelial cells *in vivo* and/or *in vitro*. Since ciprofloxacin suppressed the butyrate-mediated induction of cathelicidins, we also investigated the influence of ciprofloxacin on the induction of human β-defensins (HBDs) *in vitro*. A genome wide microarray analysis was performed in order to profile the expression of co-regulated genes. Histone modifications and phosphorylation of MAP kinases were assessed for potential regulatory mechanisms. Lastly, to evaluate cathelicidin suppression as a causal link to CDAD, the inhibitory effect of human cathelicidin LL-37 on *C. difficile* was investigated.

## 2. Results

### 2.1. Effect of Antibiotics on Expression of the CAMP Gene Encoding LL-37 in HT-29, a Colonic Epithelial Cell Line

**Figure 1:**
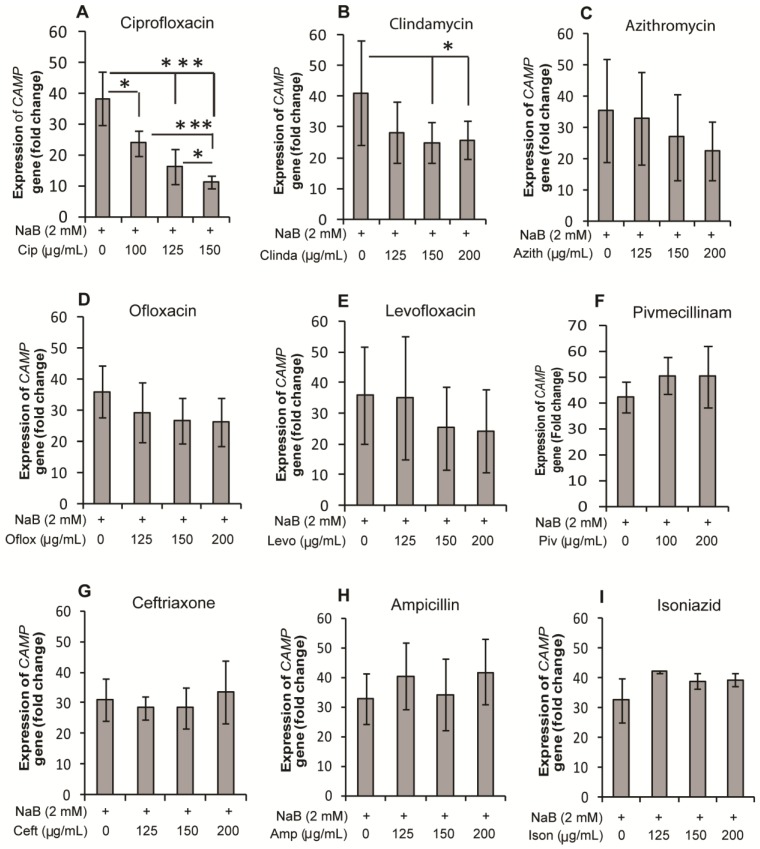
Effect of antibiotics on NaB-induced expression of the *CAMP* gene encoding LL-37 in HT-29 cells. HT-29 cells were stimulated for 24 h with 2 mM NaB alone or in combination with different concentrations of (**A**) ciprofloxacin; (**B**) clindamycin; (**C**) azithromycin; (**D**) ofloxacin; (**E**) levofloxacin; (**F**) pivmecillinam; (**G**) ceftriaxone; (**H**) ampicillin; and (**I**) isoniazid. RNA was extracted from cells and cDNA prepared, which was used to quantify *CAMP* gene (LL-37 transcript) expression by real time qPCR. *CAMP* expression is presented as fold change to control (untreated) cells. Data are given as mean ± SD of seven replicates. One way ANOVA of original data in case of clindamycin or log-transformed data for other antibiotics was utilized in comparing between different groups. Pair-wise effects between groups were compared by the Holm-Sidak *post hoc* comparison procedure. * *p* < 0.05, *** *p* < 0.001. NaB: sodium butyrate.

A selection of antibiotics, *i.e.*, ciprofloxacin, clindamycin, ofloxacin, levofloxacin, pivmecillinam, azithromycin, ceftriaxone, ampicillin, and isoniazid were screened by real-time PCR for their effect on the *CAMP* gene expression in HT-29 cells in the presence or absence of sodium butyrate (NaB). Stimulation of cells with NaB for 24 h resulted in a significant increase in *CAMP* gene expression compared to unstimulated cells (30–40 fold, *p* < 0.001). Ciprofloxacin suppressed this induction significantly (*p* < 0.05 with 100 µg/mL ciprofloxacin; *p* < 0.001 with 125 and 150 µg/mL ciprofloxacin) in a concentration dependent manner (Figure 1A). Clindamycin also exhibited significant suppression (*p* = 0.069, 0.016 and 0.028 with 125, 150 and 200 µg/mL clindamycin, respectively), although the degree of suppression was much lower than for ciprofloxacin (Figure 1B). Azithromycin, ofloxacin, and levofloxacin reduced the NaB-induced *CAMP* gene, but the effect was not significant (Figure 1C–E). Pivmecillinam, ampicillin, ceftriaxone, and isoniazid did not show any effect on the *CAMP* gene induction (Figure 1F–I). In the absence of NaB, no antibiotic had any effect on *CAMP* gene expression (data not shown). Thus, ciprofloxacin and to a lesser extent clindamycin significantly down-regulated NaB-induced *CAMP* expression, while other antibiotics exhibited no significant effect on the induction. Similar results were obtained, when the cells were stimulated for 48 h (data not shown). Notably, by trypan blue assay, no effect on cell viability was observed after the stimulation of the cells.

### 2.2. Effect of Ciprofloxacin and Pivmecillinam on the Expression of LL-37 Peptide and Its Proform hCAP18 in HT-29 Cells

**Figure 2:**
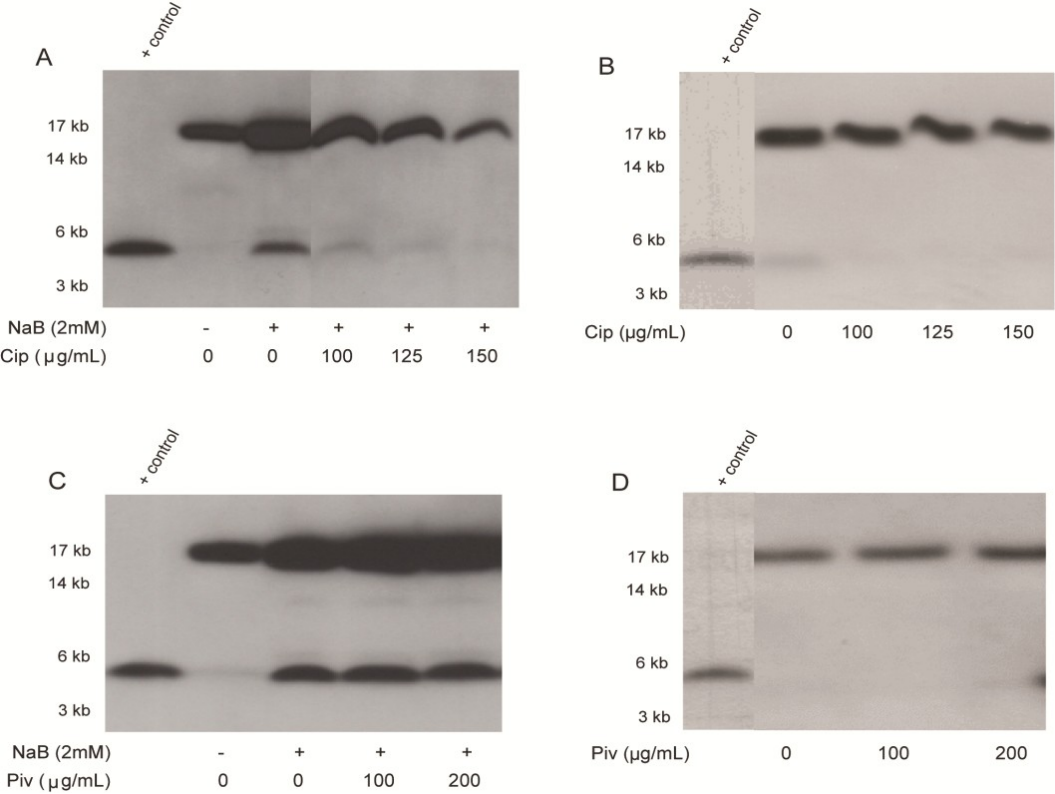
Effect of ciprofloxacin and pivmecillinam on LL-37 peptide and hCAP-18 in HT-29 cell supernatants. HT-29 cells were stimulated for 24 h with NaB and/or ciprofloxacin (**A**,**B**), with NaB and/or pivmecillinam; (**C**,**D**). Release of LL-37 peptide (lower band) and its proform hCAP-18 (upper band) in culture supernatants was detected by Western blot analysis. Representative pictures are given. Synthetic LL-37 peptide was included as positive control. The blot data in panel 2A are composites of two portions of a larger data set, while data of panels 2B and 2D are composites of two experiments. NaB: sodium butyrate; Cip: ciprofloxacin; Piv: pivmecilinam.

Since a prominent effect of ciprofloxacin was observed on transcriptional level of LL-37 expression, we further investigated the effect on the peptide/protein level by Western blot analysis. The effect of pivmecillinam, a non-responder on *CAMP* gene expression was also evaluated in parallel. Mature LL-37 peptide (4.5 kD) was either present in low level or not detected in unstimulated culture supernatant of HT-29 cells. However, after stimulation with NaB, the expression was increased and the peptide was clearly detected (Figure 2A,C). Up-regulation of the pro-form hCAP-18 (18 kD) was also observed in the culture supernatants of NaB-stimulated cells compared to unstimulated cells (Figure 2A,C). A dose-dependent down-regulation of NaB-induced expression of hCAP-18 and LL-37 was observed with ciprofloxacin (Figure 2A). Ciprofloxacin alone had no obvious effect on constitutive expression of hCAP-18 or LL-37 (Figure 2B). Pivmecillinam had no detectable effect on constitutive or NaB-induced expression of hCAP-18 or LL-37 (Figure 2C,D). These results clearly demonstrate that ciprofloxacin dose-dependently suppresses LL-37 induction by butyrate at both transcriptional and peptide/protein levels in colon epithelial cells.

### 2.3. In Vivo Effect of Ciprofloxacin and Pivmecillinam on Cathelicidin Expression in Colonic Epithelia

**Figure 3:**
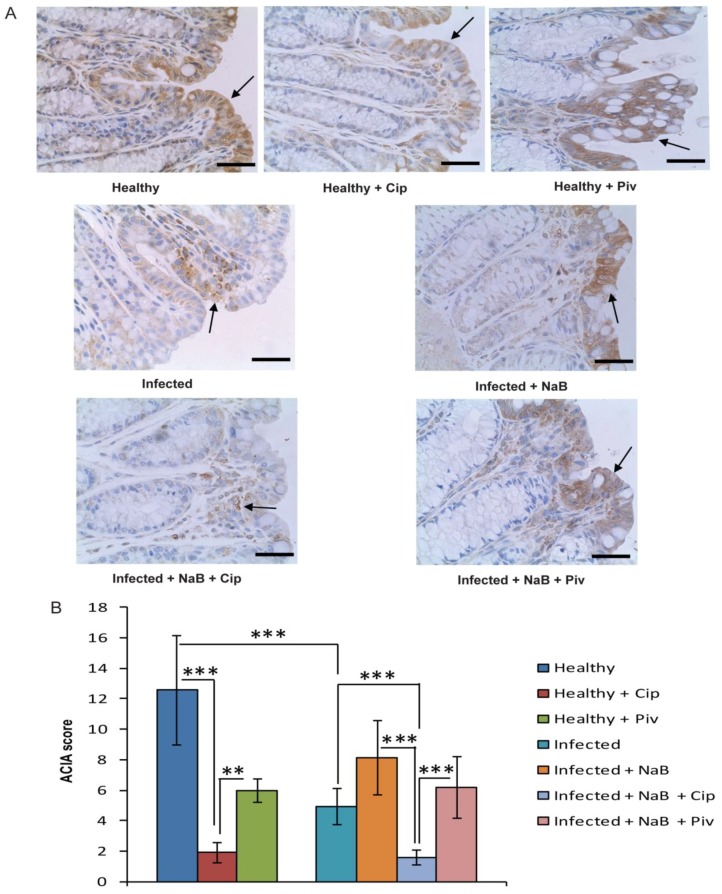
Effect of ciprofloxacin and pivmecillinam on CAP-18 expression in rectal epithelia of rabbit. Healthy rabbits were either treated with ciprofloxacin (*n* = 3) or pivmecillinam (*n* = 3) or left untreated (*n* = 3). *Shigella* infected rabbits treated with 2 mM NaB alone (*n* = 3) or together with ciprofloxacin (*n* = 3) or pivmecillinam (*n* = 3) or left untreated (*n* = 3). Mucosal sections of rectum were stained with the rabbit cathelicidin CAP-18 specific antibody. (**A**) Representative photomicrographs of CAP-18 immunostaining (arrows). Bars equal to 50 µm; (**B**) Semi-quantification of CAP-18 immunostaining are expressed as ACIA score (See materials and methods). Data are given as mean ± standard deviation. One way ANOVA of log-transformed data was utilized in comparing between different groups. Pair-wise effects between different groups were compared by the Holm-Sidak *post hoc* comparison procedure. ** *p* < 0.01, *** *p* < 0.001. NaB: sodium butyrate; Cip: ciprofloxacin; Piv: pivmecilinam.

The physiological relevance of the *in vitro* effect of ciprofloxacin and pivmecillinam on cathelicidin expression was investigated in healthy rabbits and a rabbit model of shigellosis, representing infectious diarrhoea. Immunohistochemical analysis revealed significant suppression of the rabbit cathelicidin CAP-18 expression in the rectal epithelium of healthy rabbits treated with ciprofloxacin compared to healthy untreated rabbits (*p* < 0.001) (Figure 3A,B). Notable, butyrate is present in the colon and rectum of healthy rabbits. We confirmed results from our previous study [16], demonstrating that CAP-18 expression was significantly down-regulated in rectal epithelium of rabbits infected with *Shigella flexneri* compared to healthy rabbits (*p* < 0.001) and treatment with NaB counteracted this down-regulation. Interestingly, when ciprofloxacin was given as adjunct therapy, the induction of CAP-18 was significantly suppressed (*p* < 0.001); the level was even significantly lower than that of infected rabbits (*p* < 0.001) (Figure 3A,B). These data showed that ciprofloxacin has suppressive effect on cathelicidin expression in rectal epithelia of healthy and *Shigella*-infected rabbits. The NaB-induced reappearance of CAP-18 in rectal epithelium of *Shigella*-infected rabbits was not affected by pivmecillinam and there was a significant difference between ciprofloxacin treated and pivmecillinam treated rabbits (*p* < 0.001) (Figure 3A,B). Notably, pivmecillinam treatment reduced the expression of CAP-18 to a lesser extent than ciprofloxacin in healthy rabbits (*p* < 0.01 between ciprofloxacin treated and pivmecillinam treated healthy rabbits) (Figure 3A,B). Most likely this differential reduction of CAP-18 expression reflects effects of ciprofloxacin on both the normal flora with less butyrate production and direct on epithelial cells, while pivmecillinam only affect the butyrate production mediated by the normal flora.

### 2.4. Effect of Ciprofloxacin on the Butyrate-Induced Expression of Human β-Defensins (HBD) Transcripts in HT-29 Cells

**Figure 4:**
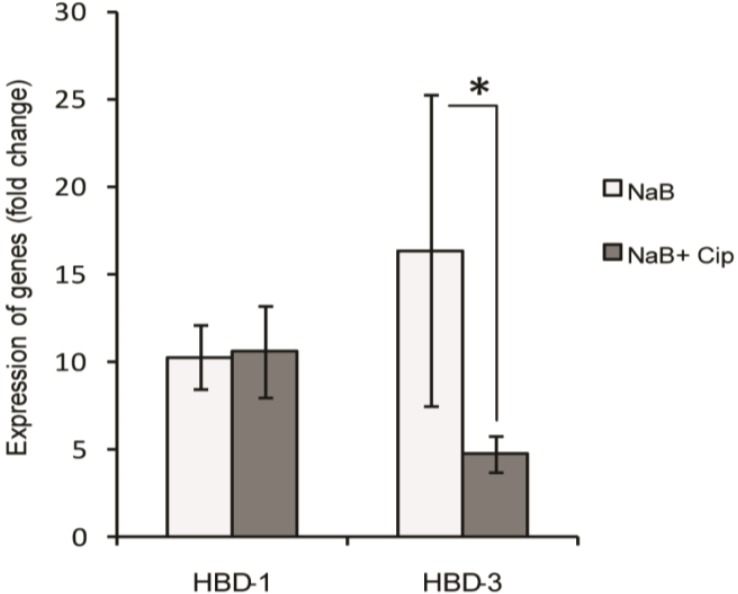
Effect of ciprofloxacin on butyrate-induced expression of HBD-1 and HBD-3 transcripts in HT-29 cells. HT-29 cells were stimulated for 24 h with 2 mM NaB alone or in combination with 150 µg/mL ciprofloxacin. RNA was extracted from cells and cDNA prepared, which was used to quantify expression of HBD-1 and HBD-3 transcripts by real time qPCR. Gene expression is presented as fold change to control (untreated) cells. Data are given as mean ± SD of four replicates. One way ANOVA of log-transformed data was utilized in comparing between different groups. Pair-wise effects between groups were compared by the Holm-Sidak *post hoc* comparison procedure. * *p* < 0.05. NaB: sodium butyrate; Cip: ciprofloxacin; HBD: human β-defensin.

We also examined the effect of ciprofloxacin on NaB-induced expression of β-defensins. Stimulation of HT-29 cells with NaB resulted in about 10 and 15 fold induction (*p* < 0.001) of the genes encoding HBD-1 and HBD-3, respectively. With the addition of 150 µg/mL ciprofloxacin, the induced expression of HBD-3 was reduced significantly (*p* = 0.019), whereas HBD-1 induction remained unaffected (Figure 4). NaB did not have any effect on the expression of the gene encoding HBD-2 (data not shown). These results suggest that ciprofloxacin also blocks butyrate-mediated induction of the gene encoding HBD-3 in colonic epithelial cells.

### 2.5. Suppressive Effect of Ciprofloxacin on Genome-Wide Expression Profile of NaB-Induced Genes in HT-29 Cells

To further investigate the suppressive effect of ciprofloxacin on NaB-induced genes, we performed a microarray analysis on RNA, extracted from HT-29 cells that were stimulated with NaB alone or in combination with ciprofloxacin. The microarray data are deposited in NCBI’s Gene Expression Omnibus and are accessible through GEO Series Accession Number GSE45220 [24]. Similar to the *CAMP* gene, the expression of several immune genes was enhanced in HT-29 cells by NaB and was subsequently suppressed by ciprofloxacin. Table 1 depicts the most interesting genes from this set that are associated with mucosal immunity including mucins, S100 calcium binding proteins and RNase A. Genes encoding the processing enzyme kallikrein, G protein coupled receptors (GPCR), interleukin receptors, interleukin 18 and nitric oxide synthase were also co-regulated with the *CAMP* gene (Table 1). The entire list of genes that were co-regulated with the *CAMP* gene is shown in Supplementary Table S1.

**Table 1:** Selected Genes, up-regulated with NaB treatment and subsequently suppressed with co-administration of ciprofloxacin.

Entrez gene ID	Gene symbol	Description	Upregulation (NaB *vs*. unstimulated)		Downregulation (NaB + Cip *vs*. NaB)	
			Fold change	*p*-value	Fold change	*p*-value
2840	GPR17	G protein-coupled receptor 17	4.37	0.0002	−2.93	0.0013
84,873	GPR128	G protein-coupled receptor 128	2.7	0.0098	−3.4	0.006
3816	KLK1	kallikrein 1	2.6	0.0012	−1.99	0.014
6035	RNASE1	ribonuclease, RNase A family, 1 (pancreatic)	8.57	0.0011	−1.91	0.042
6274	S100A3	S100 calcium binding protein A3	10.54	0.00005	−3.02	0.016
6271	S100A1	S100 calcium binding protein A1	7.72	0.0073	−2.47	0.027
57,402	S100A14	S100 calcium binding protein A14	2.59	0.0056	−1.95	0.015
4846	NOS3	nitric oxide synthase 3 (endothelial cell)	4.05	0.0002	−3.88	0.0008
143,662	MUC15	mucin 15, cell surface associated	17.2	0.03	−4.7	0.05
394,263	MUC21	mucin 21, cell surface associated	3.27	0.0024	−2.2	0.01
3606	IL18	interleukin 18 (interferon-gamma-inducing factor)	2.93	0.0013	−14.3	0.0003
400,935	IL17REL	interleukin 17 receptor E-like	2.57	0.0052	−1.96	0.011
3554	IL1R1	interleukin 1 receptor, type I	6.32	0.0071	−3.26	0.013
3557	IL1RN	interleukin 1 receptor antagonist	3.4	0.02	−3.2	0.04

NaB: sodium butyrate (2 mM); Cip: ciprofloxacin (150 µg/mL).

### 2.6. Epigenetic Modifications are Involved in the Suppressive Effect of Ciprofloxacin in HT-29 Cells

**Figure 5:**
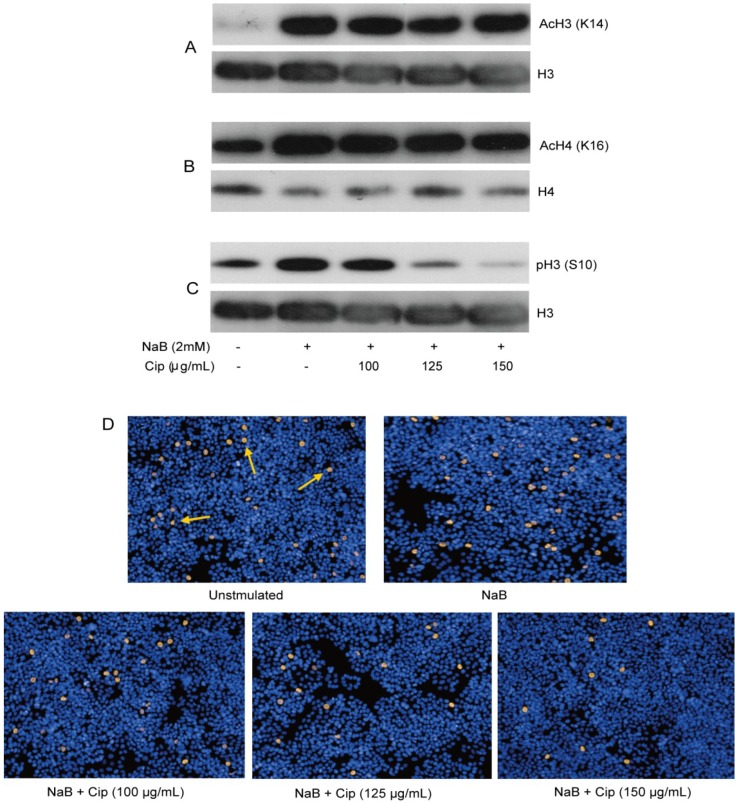
Histone modifications in HT-29 cells after treatment with NaB alone or in combination with ciprofloxacin. HT-29 cells were stimulated for 24 h with 2 mM NaB alone or in combination with different concentrations of ciprofloxacin. Histone was extracted from cells and utilized for Western blot analysis to detect (**A**) acetylation of histone H3 Lys14; (**B**) acetylation of histone H4 Lys16; (**C**) phosphorylation of histone H3 Ser10; (**D**) Phosphorylation of histone H3 at Ser10 was also detected by immunofluorescence staining of the cells. Arrows indicate examples of positively stained cells. NaB: sodium butyrate; Cip: ciprofloxacin; AcH3(K14): Acetylation of histone H3 at Lys14; AcH4(K16): Acetylation of histone H4 at Lys16; pH3(S10): Phosphorylation of histone H3 at Ser10.

Butyrate and phenylbutyrate are histone deacetylase inhibitors (HDACi) and have been demonstrated to induce *CAMP* gene expression [25,26,27]. To investigate potential epigenetic effects of NaB and/or ciprofloxacin in HT-29 cells, we evaluated acetylation of histone H3 and H4 by Western blot analysis of histone extracts. Phosphorylation of histone H3 has also been implicated in the induced expression of several genes such as c-fos, c-jun, additional activator protein-1 (AP-1) family genes and c-myc [28]. Hence, phosphorylation of H3 was also assessed by Western blot analysis of histone extracts. NaB augmented acetylation of histone H3 at Lys14, histone H4 at Lys16 and phosphorylation of histone H3 at Ser10 within 2 h of stimulation that lasted up to 24 h (Figure 5A–D). Ciprofloxacin exhibited no effect on the NaB-induced acetylation of H3 and H4 (Figure 5A,B). Interestingly, ciprofloxacin dose-dependently reduced the induction of phosphorylation of histone H3 at Ser10 (Figure 5C). Changes of H3 phosphorylation in HT-29 after treatment with NaB alone or in combination with ciprofloxacin was confirmed by the immunofluorescence staining of cells (Figure 5D). Ciprofloxacin alone had no effect on histone phosphorylation and acetylation (data not shown). These findings indicate the involvement of both acetylation and phosphorylation of histones in butyrate-induced genes. However, only phosphorylation of histone H3 was correlated with the ciprofloxacin-mediated down-regulation of butyrate-induced genes.

To approach the involvement of MAP kinase signaling pathway, the phosphorylation of ERK and p38 in HT-29 cells was investigated after treatment of the cells with NaB and/or ciprofloxacin. By Western blot analysis of cell lysates, no differences between treatment groups were observed at any time point starting from 5 min to 24 h (data not shown).

### 2.7. In Vitro Effect of Synthetic LL-37 Peptide on C. Difficile

**Figure 6:**
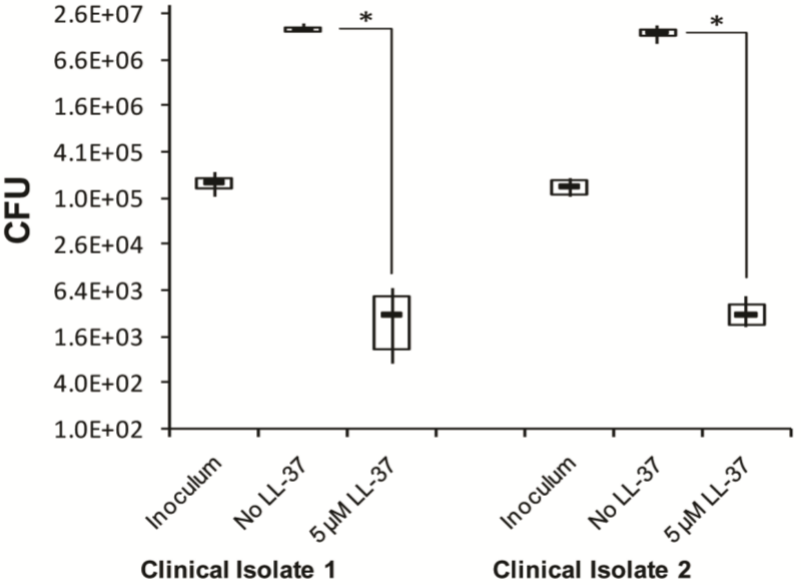
Inhibitory effect of synthetic LL-37 peptide on clinical isolates of *Clostridium difficile*. Two clinical isolates of *C. difficile* that were metronidazole and vancomycin sensitive but resistant to ciprofloxacin were treated with or without LL-37 peptide in MHB in wells of microtiter plate. Bacterial suspensions from individual wells were plated on blood-agar for counting of CFU. Lower and upper boundaries of the boxes and the horizontal bars in between indicate 25th percentile, 75th percentile and group median, respectively. Vertical lines through the boxes join the minimum and maximum values. Kruskal-Wallis ANOVA on Ranks of original data was utilized in comparing between different groups. Tukey test was used to compare the pair wise effects between treatment groups. * *p* < 0.05. CFU: colony forming unit; MHB: Mueller-Hinton broth.

To investigate if the clinical observation that ciprofloxacin causing overgrowth of *C. difficile* might be associated with inhibitory effect on cathelicidin expression in colonic epithelia, the antibacterial activity of LL-37 against *C. difficile* was evaluated. Incubation of two clinical isolates of ciprofloxacin resistant *C. difficile* with 5 µM LL-37 led to four log reduction of colony forming unit (CFU) compared to that obtained by incubating without LL-37 (*p* < 0.05) (Figure 6), showing that LL-37 is able to kill *C. difficile in vitro.*

## 3. Discussion

In this study, we demonstrate that ciprofloxacin and clindamycin significantly suppress butyrate-mediated induction of cathelicidin in the colon epithelial cell line HT-29. Suppressed expression of cathelicidin by ciprofloxacin was also observed *in vivo* in the rectal epithelium of healthy rabbits, where butyrate is present, and butyrate-treated *Shigella*-infected rabbits. Moreover, ciprofloxacin suppressed butyrate-induced expression of HBD-3 in HT-29 cells. The direct effect of ciprofloxacin *in vivo* is demonstrated in healthy rabbits by higher suppression of CAP-18 by ciprofloxacin as compared to pivmecillinam. Both antibiotics disrupt the normal flora, leading to reduced butyrate level and lower CAP-18 expression. However, ciprofloxacin exerts additional suppression by directly affecting epithelial cells. The induction of the two peptides by butyrate and subsequent reduction by ciprofloxacin is correlated to the phosphorylation status of histone H3 at Ser10. Furthermore, the peptide LL-37 exhibited inhibitory effect on the growth of two ciprofloxacin resistant strains of *C. difficile in vitro.*

Ciprofloxacin, a second generation fluoroquinolone antibiotic, has a broad spectrum bactericidal activity and acts by inhibiting bacterial DNA gyrase/topoisomerase. Several antibiotics including ciprofloxacin and other fluoroquinolones are known to exert modulatory effects on immunity such as down-regulation of proinflammatory cytokines [22,29]. In addition, ciprofloxacin modulates phagocytic and killing capacity of neutrophils and macrophages [30,31], and affects the expression of toll-like receptors, CD14, CD40 and intracellular adhesion molecules in monocytes [32,33]. Furthermore, ciprofloxacin inhibits cytokine-induced nitric oxide production in human colon epithelial cells [34]. Here, we have shown that ciprofloxacin dose-dependently inhibits butyrate-induced expression of the human cathelicidin LL-37 and β-defensin (HBD)-3 in HT-29 cells. Our study indicates that the general mechanism on gene expression by ciprofloxacin is of epigenetic character through reduced phosphorylation of histone H3 that may explain the broad effects described for this antibiotic.

We did not detect any effect on LL-37 expression by ciprofloxacin in HT-29 cells in the absence of butyrate. This could be in agreement with previous observations that quinolones only exert immunomodulatory effects in the presence of co-stimulant(s) such as endotoxin, cytokines or stress [32,33,34,35]. Butyrate is constantly produced in the large intestine by the fermentation of dietary fibers, which coincides with high expression of CAP-18 and LL-37 in the rectal epithelia of healthy rabbits and humans, respectively (Figure 3 and references [16,36]). This *in vivo* induced expression of CAP-18 by butyrate in the rectal epithelium of healthy rabbits was also suppressed by ciprofloxacin. In addition, ciprofloxacin inhibited the counteracting effect of butyrate on CAP-18 expression in the rabbit model of shigellosis.

*In vitro* suppression of LL-37 clearly demonstrates the direct effect of ciprofloxacin on host epithelial cells. It is conceivable that the *in vivo* reduction of CAP-18 by ciprofloxacin is also a direct effect on rectal epithelial cells, not only an effect caused by killing of the microbiota. On the other hand, pivmecillinam had no suppressive effect on CAP-18 expression in epithelial cells *in vitro*, but reduced the cathelicidin levels in healthy rabbits, indicating a secondary effect due to less butyrate production by the normal flora.

Ofloxacin and levofloxacin, second and third generation of flouroquinolones, respectively, did not significantly suppress the butyrate-induced LL-37 expression in HT-29 cells. The difference in the degree of suppression might be attributed to structural differences of these fluoroquinolones. Ciprofloxacin possesses a cyclopropyl ring at the quinolone ring, but ofloxacin and levofloxacin lack this ring. Quinolones having cyclopropyl ring were shown to have enhanced anti-leukaemic and haematopoietic effects, as opposed to the quinolones lacking this ring [37,38].

Clindamycin, a lincosamide antibiotic, at higher concentrations than ciprofloxacin significantly suppressed the induction of LL-37 by butyrate in HT-29 cells. In contrast, azithromycin, pivmecillinam, ampicillin, ceftriaxone, and isoniazid exhibited no significant suppression. These results show that the suppressive effect observed is specific for ciprofloxacin and clindamycin, indicating also a direct effect of these antibiotics on host cells and not only mediated by the disturbance of the microbiota composition.

Numerous additional genes were up-regulated in HT-29 cells after stimulation with NaB as revealed by microarray analysis, emphasizing LL-37 as a marker for mucosal immunity. Interestingly, many of these butyrate-induced genes were down-regulated by ciprofloxacin and are linked to innate immunity, including S100 calcium binding proteins, RNase A, mucins, a cathelicidin processing enzyme (kallikrein) and G protein coupled receptors (GPCR). Calgranulins (S100A8, S100A9 and S100A12) and calprotectin (heterodimer of S100A8 and S100A9) belonging to the family of S100 calcium binding proteins, which exhibit antimicrobial and immunomodulatory properties [39]. Some members of the RNase A superfamily are also involved in host defense [40]. Members of the kallikrein family were shown to process hCAP18 into LL-37 followed by subsequent cleavage to modify the activity of LL-37 in skin [41]. Interestingly, it was recently reported that doxycycline inhibits kallikrein-related peptidases and thus inhibits the generation of active LL-37 [42]. Members of G protein coupled receptors (GPCR) have been shown to mediate the function of short chain fatty acids including butyrate [43,44]. GPCR might have interesting implications on butyrate- and/or antibiotics-mediated AMP expression in colonic epithelium. Thus, our results demonstrate a broad effect of ciprofloxacin on the expression of innate immune genes that may promote secondary infections.

Western blot analysis of histone extracts of HT-29 cells revealed a rapid and persistent increase of phosphorylation and acetylation of histone H3 at Ser10 and Lys14, respectively, after stimulation of cells with NaB. Hyperacetylation was also found at Lys16 of histone H4. Notably, ciprofloxacin, at all time points (2 h, 4 h, 6 h and 24 h), dose-dependently suppressed the induced phosphorylation of histone H3, which was confirmed by immunofluorescence staining of the cells. However, ciprofloxacin did not affect the induced acetylation of histones. These findings imply epigenetic modifications as part of the ciprofloxacin mediated suppression of butyrate-induced gene expression. We have earlier shown that cyclohexamide, an inhibitor of protein synthesis, blocked the *CAMP* gene induction by NaB [27]. Therefore we propose that general histone modification(s) allow expression of genes encoding regulatory proteins for the *CAMP* gene.

Impaired immune responses have been reported as important factors for CDAD, apart from the disruption of the normal gut flora [45]. Here, we have demonstrated that LL-37 exhibits antibacterial activity against two clinical isolates of *C. difficile in vitro*, which suggests that suppression of AMPs after antibiotic treatment might favor the overgrowth of *C. difficile*. In a recent study, intra-colonic administration of mCRAMP to *C. difficile*-infected mice improved toxin A-mediated colitis outcome [46]. α-defensins were also shown to inhibit the cytotoxic effect of *C. difficile* toxin B [47]. Moreover, in a previous study, down-regulation of the antimicrobial protein RegIIIγ in the small intestinal mucosa of antibiotic-treated mice was shown to increase the colonization of gut by vancomycin-resistant Enterococcus [48]. These findings indicate that suppression of AMPs may facilitate infection of the gut mucosa by enteropathogens, such as *C. difficile* and resultant disease manifestation. However, *C. difficile* infection in wild type and CRAMP^−/−^ mice demonstrated similar colonic inflammation in both group of mice [46]. Since, mice were pretreated with antibiotic cocktail to induce *C. difficile* infection, it is possible that mCRAMP was down-regulated by the antibiotics in the colonic epithelium of wild type mice, favoring *C. difficile* infection. Moreover, Salzman *et al.* has shown that enteric defensins regulate the composition of the intestinal flora [12], suggesting a critical role of AMPs in intestinal homeostasis. Hence, by suppressing AMPs, antibiotic treatment of ciprofloxacin or clindamycin may further contribute to alteration of the microbial niche, promoting CDAD.

## 4. Experimental

### 4.1. Ethics Statement

Experiment in animal model (Research protocol # 2007-065) was approved by the Animal Experimentation Ethics Committee (AEEC) of the International Centre for Diarrhoeal Disease Research, Bangladesh (icddr,b) on May 07, 2008. All experiments conformed to the rules and guidelines of icddr,b, which was developed based on the recommendations in the Guide for the Care and Use of Laboratory Animals of the National Institutes of Health (NIH). Animal experimentation in this study complied with the “3Rs”. REPLACEMENT: Live animals had to be used to evaluate the physiological relevance of the *in vitro* effect of ciprofloxacin and pivmecillinam on cathelicidin expression. REDUCTION: Minimum numbers of animals were used. REFINEMENT: Rabbits were kept in individual cages and provided with food and water ad libitum. Rabbits were sacrificed within a very short period with an overdose of intravenous sodium pentobarbital (66 mg per kg body weight). Appropriate veterinary care was taken for assessing and preventing pain and distress by expert veterians of the animal facility.

### 4.2. Peptides, Antibodies, Antibiotics and Sodium Butyrate

Synthetic bioactive peptides LL-37 (LLGDFFRKSKEKIGKEFKRIVQRIKDFLRNLVPRTES) and CAP-18 (GLRKRLRKFRNKIKEKLKKIGQKIQGLLPKLAPRTDY), and affinity-purified chicken anti-CAP-18 antibody were purchased from Innovagen (Lund, Sweden). Monoclonal antiserum against LL-37 was developed in mouse hybridoma cells [49]. Sodium butyrate (NaB), ciprofloxacin, pivmecillinam, ofloxacin, levofloxacin, azithromycin, clindamycin, ampicillin, ceftriaxone and isoniazid were purchased from Sigma-Aldrich (St Louis, MO, USA). Rabbit polyclonal antibodies to phospho-histone H3 (S10) or acetyl-histone H3 (K14) and mouse monoclonal antibodies to histone H3 or H4 were from Abcam (Cambridge, UK). The source of polyclonal antibody to acetyl-histone H4 (K16) was Active Motif (La Hulpe, Belgium). Rabbit polyclonal antibodies to phospho-ERK and phospho-p38 were purchased from Cell signaling technology Inc. (Danvers, MA, USA).

### 4.3. Cell Line and Growth Conditions

HT-29 (ATCC, HTB-38), a human colonic epithelial cell line, was maintained in RPMI-1640 supplemented with 10% fetal calf serum (FCS), 25 mM HEPES, 2 mM l-glutamine and penicillin-streptomycin (PEST) (Life Technologies, New York, NY, USA) at 37 °C in 5% CO_2_.

### 4.4. Primers

The sequences of the primers for real time qPCR were: CAMP (LL-37 transcript), forward 5'-TCACCAGAGGATTGTGACTTCAAC-3' and reverse 5'-TGAGGGTCACTGTCCCCATAC-3'; human β-defensin (HBD) -1 transcript, forward 5'-ATGGCCTCAGGTGGTAACTTTC-3' and reverse 5'-CACTTGGCCTTCCCTCTGTAAC-3'; HBD-2 transcript, forward 5'-GCCTCTTCCAGGTGTTTTTG-3' and reverse 5'-GAGACCACAGGTGCCAATTT-3'; HBD-3 transcript, forward 5'-GCTGCCTTCCAAAGGAGGA-3' and reverse 5'-TTCTTCGGCAGCATTTTCG-3'.

### 4.5. Bacterial Strains

A clinical isolate of *Shigella flexneri* 2a, isolated from patient’s stool at Dhaka Hospital of icddr,b was used to infect rabbits [16]. For the *in vitro* killing assay, two strains of *Clostridium difficile*, isolated from patients stool at Karolinska University Hospital, Huddinge, Stockholm, Sweden were used. These two clinical isolates of *C. difficile* were metronidazole and vancomycin sensitive but resistant to ciprofloxacin. All these bacterial strains were isolated from routine clinical stool samples and these clinical isolates are anonymous.

### 4.6. Stimulation of Cells

HT-29 Cells, grown up to 80%–90% confluence in tissue culture plates (Corning, Steuben County, NY, USA), were stimulated with 2 mM NaB and/or with different concentrations of several antibiotics for 24 or 48 h. Stimulations were performed in culture medium in the absence of FCS and PEST. Cells incubated with only culture medium served as negative control. The viability of cells was checked by trypan blue assay. To enrich peptides/proteins in culture supernatants, trifluoroacetic acid (TFA) was added to supernatants and were applied to acetonitrile (AcN)-activated OASIS cartridges (Waters, Milford, MA, USA) equilibrated in aqueous 0.1% TFA. Bound peptides/proteins were eluted with 80% aqueous AcN in 0.1% TFA and lyophilized. RNA was extracted from cells utilizing RNeasy RNA purification kit according to the manufacturer’s instruction (Qiagen GmbH, Hilden, Germany). Corresponding cDNA was synthesized using a reverse transcriptase kit (Biorad Laboratories Inc., Berkeley, CA, USA).

### 4.7. Real-Time RT-PCR

cDNA samples from HT-29 cells were used to measure the level of the *CAMP* gene and genes encoding HBD-1, HBD-2, or HBD-3 relative to the housekeeping gene 18S rRNA. Measurements were performed by SYBRGreen based real-time quantitative RT-PCR, using a CFX-96 real time system instrument (Biorad). Results were expressed as fold changes in the treated cells compared to control cells.

### 4.8. Microarray Analysis

Quality and integrity of RNA from HT-29 cells were assessed using the Agilent Bioanalyzer 2100 (Agilent Technologies, Santa Clara, CA, USA). Individual samples were hybridized to Affymetrix Human Gene 1.1 ST arrays. The arrays were scanned using the GeneTitan scanner. Probe cell intensity (CEL) files were preprocessed in Affymetrix Expression Console (EC, version 1.1; Santa Clara, CA, USA) using the following methods; (i) summarization: Probe Logarithmic Intensity Error (PLIER); (ii) background correction: Perfect Match probes-Guanine, Cytosine composition-based background correction (PM-GCBG); (iii) normalization: Global Median. Expression levels between control and treatment groups and between treatment groups were compared using two-tailed, Student’s *t*-test.

### 4.9. Cell Lysis and Histone Extraction

Stimulated/unstimulated HT-29 cells were lysed with RIPA buffer (Sigma-Aldrich). After centrifugation at 13,000 rpm for 10 min at 4 °C, cell lysates were collected and the remaining cell pellets were used for histone extraction following the method of Lee *et al.* with some modifications [50]. Briefly, cell pellets were washed twice with PBS and resuspended in ice-cold 0.4 M H_2_SO_4_. Pellets were broken down with brief sonication and incubated overnight on a shaker at 4 °C. After centrifugation at 13,000 rpm for 1 h at 4 °C, supernatants were collected and histones were precipitated with ice-cold acetone overnight at −20 °C. The resulting translucent pellets of histones after centrifugation at 13,000 rpm for 10 min at 4 °C were air dried, resuspended in ice-cold water and used for Western blot analysis.

### 4.10. Western Blot Analysis

Discontinuous sodium dodecylsulfate-polyacrylamide gel electrophoresis (SDS-PAGE), employing 4%–12% NuPAGE Ready Gels (Life Technologies) followed by Western blot analysis were utilized for detection of LL-37 in cell culture supernatants, phosphorylation of ERK and p38 in cell lysates, and phosphorylation/acetylation of histones in histone extracts. After electrophoretic separation, materials in the gel were blotted onto polyvinyldifluoride (PVDF) membrane (Life Technologies) by electrophoretic transfer. Immunoreactivity was detected by subsequent incubation of the membrane with specific primary antibodies and corresponding secondary antibodies conjugated with horseradish peroxidase (Jackson Immunoresearch Laboratories Inc., West Grove, PA, USA). The enhanced chemiluminescence (ECL) Western blotting detection system (GE Healthcare UK Ltd., Buckinghamshire, UK) was utilized to visualize protein/peptide bands.

### 4.11. Immunofluorescence Staining of Phosphorylated Histone H3 at Ser10

HT-29 cells were stimulated with NaB and/or ciprofloxacin in 96 well tissue culture imaging plate (BD Biosciences, Woburn, MA, USA). Cells were fixed with 2% paraformaldehyde followed by permeabilization with 0.1% triton-X-100 (ICN Biomedicals, Solon, OH, USA). Cells were then subsequently incubated with rabbit polyclonal antibody for phospho-histone H3 (S10) and anti-rabbit antibody conjugated with AlexaFluor 594 (Life Technologies). DAPI, a nuclear die, was added to the cells and immunofluorescence staining was detected in an Operetta image analyzer (Perkin Elmer Inc., Alameda, CA, USA).

### 4.12. Rabbit Model

Inbred New Zealand white rabbits (Charles River Laboratories, Wilmington, MA, USA) of either sex, aged between 2.5–3 months and weighing 1.8–1.9 kg were maintained in the animal resource facilities of icddr,b. Healthy rabbits, free of enteric pathogens (e.g., *Salmonella*, *Shigella*, and *Vibrio cholera*) and coccidia were studied. Twelve rabbits were infected with *Shigella flexneri* 2a preceded by 36 h of starvation. Bacterial suspension [109 CFU in 7 ml of normal saline (0.9% wt/vol, pH 7.2)] was given via sterile orogastric feeding tube to each rabbit. Rabbits developed dysentery within 24 h of bacterial inoculation. The infected rabbits were orally treated with NaB (0.14 mmol/kg body weight/dose) (*n* = 3), or NaB in combination with ciprofloxacin/pivmecillinam (20 mg/kg body weight/dose) (*n* = 3 per antibiotic group) in 20 mM sodium chloride (pH 7.2) by utilizing sterile feeding tubes. The treatments were given twice daily (at around 10 am and 4 pm) for three consecutive days. The remaining three infected rabbits were kept untreated. Healthy rabbits were either treated with ciprofloxacin (*n* = 3) or pivmecillinam (*n* = 3) or left untreated (*n* = 3). When the treatment regime was over, all rabbits were sacrificed with an overdose of intravenous sodium pentobarbital (66 mg/kg body weight) (Sigma-Aldrich). The abdomen of each sacrificed rabbit was opened and sections of rectum were collected in 10% buffered formalin and utilized for immunohistochemical evaluation.

### 4.13. In Situ Immunohistochemical Staining and Quantification of CAP-18 Peptide/Protein Expression in Rectal Mucosa of Rabbits

Formalin fixed tissue pieces of rectum were embedded in paraffin and cut into three micron thick sections. Sections were deparaffinized, and stained with CAP-18 antibody (6.8 µg/mL) and corresponding secondary antibody. Immunohistochemical staining of CAP-18 was analyzed by using a microscope (Leica Microsystems GmbH, Wetzlar, Germany) and the image analysis system Quantimate Q550 (Leica). CAP-18 staining were quantified in the epithelial areas in each tissue section and the results were given as ACIA (Acquired Computerized Image Analysis) score, *i.e*., total positively stained area x total mean intensity (1–256 levels per pixel) of the positive area divided by total cell area [51].

### 4.14. In Vitro Bacterial Killing

LL-37 peptide (5 µM) was added to the isolates (see under bacterial strains) of *C. difficile* (10^5^ CFU) in Mueller-Hinton broth (MHB; Becton Dickinson, NJ, USA) in wells of microtiter plate (Nunc, Thermo Fisher Scientific, NY, USA) in a final volume of 200 µL. Control wells contained MHB alone and bacteria in MHB without LL-37. The plate was incubated for 48 h in an anaerobic jar at 37 °C. Bacterial suspension/media from individual wells were plated on blood-agar for counting of CFU.

### 4.15. Statistical Analyses

Statistical analyses were performed by using Sigma STAT for Windows version 3.1 and SPSS. Data were expressed as mean ± standard deviation or median with 25th and 75th percentiles. One way ANOVA was utilized in comparing effects between treatment groups. Data that were not normally distributed and/or failed the equal variance test were log transformed before ANOVA analysis. When significant effect was found between treatment groups, the pair-wise effects between treatment groups were compared by the Holm-Sidak *post hoc* comparison procedure. If the normality and/or equal variance test failed even after log-transformation, original data were analyzed by Kruskal-Wallis ANOVA on Ranks. After getting significant effect between treatment groups, the Tukey test was used to compare the pair-wise effects between treatment groups. Probabilities were regarded as significant when *p* < 0.05.

## 5. Conclusions

Our study shows that ciprofloxacin and clindamycin significantly downregulate butyrate-mediated induction of innate immune components in the large intestinal epithelia. The prevailing explanation for CDAD pathogenesis is that antibiotic treatment disturbs the normal flora. Here, we demonstrate that the antibiotic-mediated suppression of innate immune effectors might also contribute to CDAD.

## Supplementary Files

Supplementary File 1
