# Supplementary material for: Ciprofloxacin Affects Host Cells by Suppressing Expression of the Endogenous Antimicrobial Peptides Cathelicidins and Beta-Defensin-3 in Colon Epithelia
Source: Antibiotics (Basel). 2014 Jul 25;3(3):353–74. doi: 10.3390/antibiotics3030353 (PMC4790365; doi:10.3390/antibiotics3030353)
Supplement: Supplementary File 1 [file antibiotics-03-00353-s001.pdf]

# Supplementary Material

**Table S1.** Genes, upregulated with NaB treatment and subsequently suppressed with co-administration of ciprofloxacin.

| Entrez gene ID | Gene symbol | Description | Upregulation (NaB *vs*. unstimulated) | | Downregulation (NaB + cip *vs*. unstimulated) | |
|---|---|---|---|---|---|---|
| | | | Fold change | *p*-value | Fold change | *p*-value |
| 6300 | MAPK12 | mitogen-activated protein kinase 12 | 3.61 | 0.0048 | −2.18 | 0.016 |
| 394263 | MUC21 | mucin 21, cell surface associated | 3.27 | 0.0024 | −2.2 | 0.01 |
| 57678 | GPAM | glycerol-3-phosphate acyltransferase, mitochondrial | 2.78 | 0.001 | −2.42 | 0.0016 |
| 66002 | CYP4F12 | cytochrome P450, family 4, subfamily F, polypeptide 12 | 2.31 | 0.0017 | −4.22 | 0.0002 |
| 2941 | GSTA4 | glutathione S-transferase alpha 4 | 2.28 | 0.015 | −4.24 | 0.0021 |
| 290 | ANPEP | alanyl (membrane) aminopeptidase | 5.12 | 0.004 | −7.41 | 0.003 |
| 25809 | TTLL1 | tubulin tyrosine ligase-like family, member 1 | 2.03 | 0.019 | −3.71 | 0.0022 |
| 23563 | CHST5 | carbohydrate (N-acetylglucosamine 6-O) sulfotransferase 5 | 2.6 | 0.0078 | −10.33 | 0.0014 |
| 2875 | GPT | glutamic-pyruvate transaminase (alanine aminotransferase) | 2.05 | 0.00002 | −2.42 | 0.0005 |
| 6274 | S100A3 | S100 calcium binding protein A3 | 10.54 | 0.00005 | −3.02 | 0.016 |
| 2840 | GPR17 | G protein-coupled receptor 17 | 4.37 | 0.0002 | −2.93 | 0.0013 |
| 4846 | NOS3 | nitric oxide synthase 3 (endothelial cell) | 4.05 | 0.0002 | −3.88 | 0.0008 |
| 574029 | DUSP5P | dual specificity phosphatase 5 pseudogene | 8.51 | 0.0005 | −2.8 | 0.0024 |
| 7106 | TSPAN4 | tetraspanin 4 | 3.51 | 0.0006 | −2 | 0.015 |
| 57190 | SEPN1 | selenoprotein N, 1 | 2.92 | 0.0009 | −2.02 | 0.0044 |
| 6035 | RNASE1 | ribonuclease, RNase A family, 1 (pancreatic) | 8.57 | 0.0011 | −1.91 | 0.042 |
| 3816 | KLK1 | kallikrein 1 | 2.6 | 0.0012 | −1.99 | 0.014 |
| 3606 | IL18 | Interleukin 18 (interferon-gamma-inducingfactor) | 2.93 | 0.0013 | −14.3 | 0.0003 |
| 222 | ALDH3B2 | aldehyde dehydrogenase 3 family, member B2 | 5.91 | 0.0014 | −5.36 | 0.0016 |
| 51148 | CERCAM | cerebral endothelial cell adhesion molecule- | 9.17 | 0.0016 | −2.2 | 0.026 |
| 54763 | ROPN1 | ropporin, rhophilin associated protein 1 | 3.98 | 0.0016 | −2.69 | 0.0044 |
| 250 | ALPP | alkaline phosphatase, placental (Regan isozyme) | 3.64 | 0.0016 | −2.26 | 0.0011 |
| 79908 | BTNL8 | butyrophilin-like 8 | 2.83 | 0.0017 | −2.47 | 0.0039 |
| 23017 | FAIM2 | Fas apoptotic inhibitory molecule 2 | 3.54 | 0.0017 | −2.23 | 0.01 |
| 101 | ADAM8 | ADAM metallopeptidase domain 8 | 2.4 | 0.0017 | −2.17 | 0.0054 |

**Table S1.** *Cont.*

| Entrez gene ID | Gene symbol | Description | Upregulation (NaB *vs.* unstimulated) | | Downregulation (NaB + cip *vs.* unstimulated) | |
|---|---|---|---|---|---|---|
| | | | Fold change | *p*-value | Fold change | *p*-value |
| 54866 | PPP1R14D | protein phosphatase 1, regulatory (inhibitor) subunit 14D | 4.29 | 0.0019 | −3.38 | 0.0051 |
| 2696 | GIPR | gastric inhibitory polypeptide receptor | 3.8 | 0.0019 | −2.08 | 0.0079 |
| 10347 | ABCA7 | ATP-binding cassette, sub-family A (ABC1), member 7 | 3.49 | 0.002 | −2.54 | 0.0047 |
| 3759 | KCNJ2 | potassium inwardly-rectifying channel, subfamily J, member 2 | 2.1 | 0.002 | −2.88 | 0.005 |
| 23546 | SYNGR4 | synaptogyrin 4 | 3.44 | 0.0026 | −2.22 | 0.033 |
| 284422 | C19orf77 | chromosome 19 open reading frame 77 | 4.62 | 0.0028 | −4.1 | 0.0033 |
| 147920 | IGFL2 | IGF-like family member 2 | 4.14 | 0.0031 | −2.1 | 0.018 |
| 4241 | MFI2 | antigen p97 (melanoma associated) identified by monoclonal antibodies 133.2 and 96.5 | 5.11 | 0.0032 | −3.9 | 0.004 |
| 152015 | ROPN1B | ropporin, rhophilin associated protein 1B | 4.46 | 0.0032 | −2.9 | 0.006 |
| 9056 | SLC7A7 | solute carrier family 7 (cationic amino acid transporter, y + system), member 7 | 2.17 | 0.0036 | −2 | 0.022 |
| 2774 | GNAL | guanine nucleotide binding protein (G protein), alpha activating activity polypeptide, olfactory type | 2.58 | 0.0041 | −3.58 | 0.0017 |
| 9429 | ABCG2 | ATP-binding cassette, sub-family G (WHITE), member 2 | 2.6 | 0.0043 | −2.56 | 0.003 |
| 5118 | PCOLCE | procollagen C-endopeptidase enhancer | 7.92 | 0.0044 | −2.5 | 0.03 |
| 79628 | SH3TC2 | SH3 domain and tetratricopeptide repeats 2 | 3.1 | 0.0048 | −2.9 | 0.013 |
| 7079 | TIMP4 | TIMP metallopeptidase inhibitor 4 | 2.64 | 0.0049 | −2.08 | 0.004 |
| 400935 | IL17REL | interleukin 17 receptor E-like | 2.57 | 0.0052 | −1.96 | 0.011 |
| 402778 | LOC402778 | CD225 family protein FLJ76511 | 4.4 | 0.0056 | −4.55 | 0.005 |
| 57402 | S100A14 | S100 calcium binding protein A14 | 2.59 | 0.0056 | −1.95 | 0.015 |
| 2593 | GAMT | guanidinoacetate N-methyltransferase | 6.79 | 0.0057 | −6.39 | 0.006 |
| 727936 | GXYLT2 | glucosidexylosyltransferase 2 | 2.16 | 0.0059 | −2.41 | 0.0019 |
| 27120 | DKKL1 | dickkopf-like 1 (soggy) | 5 | 0.0067 | −3.6 | 0.01 |
| 3554 | IL1R1 | interleukin 1 receptor, type I | 6.32 | 0.0071 | −3.26 | 0.013 |
| 6271 | S100A1 | S100 calcium binding protein A1 | 7.72 | 0.0073 | −2.47 | 0.027 |

**Table S1.** *Cont.*

| Entrez gene ID | Gene symbol | Description | Upregulation (NaB *vs.* unstimulated) | | Downregulation (NaB + cip *vs.* unstimulated) | |
|---|---|---|---|---|---|---|
| | | | Fold change | *p*-value | Fold change | *p*-value |
| 241 | ALOX5AP | arachidonate 5-lipoxygenase-activating protein | 2.96 | 0.0075 | −2.36 | 0.01 |
| 574029 | DUSP5P | dual specificity phosphatase 5 pseudogene | 4.24 | 0.0075 | −2.61 | 0.018 |
| 79258 | MMEL1 | membrane metallo-endopeptidase-like 1 | 2.82 | 0.0075 | −3.67 | 0.008 |
| 1551 | CYP3A7 | cytochrome P450, family 3, subfamily A, polypeptide 7 | 3.19 | 0.0076 | −3.49 | 0.004 |
| 23563 | CHST5 | carbohydrate (N-acetylglucosamine 6-O) sulfotransferase 5 | 2.6 | 0.0078 | −10.33 | 0.001 |
| 2548 | GAA | glucosidase, alpha; acid | 2.8 | 0.0083 | −3.07 | 0.005 |
| 954 | ENTPD2 | ectonucleoside triphosphate diphosphohydrolase 2 | 3.6 | 0.0085 | −2.4 | 0.021 |
| 63924 | CIDEC | cell death-inducing DFFA-like effector c | 4.3 | 0.0085 | −4.5 | 0.009 |
| 64097 | EPB41L4A | erythrocyte membrane protein band 4.1 like 4A | 2.5 | 0.0089 | −3.3 | 0.006 |
| 1556 | CYP2B7P1 | cytochrome P450, family 2, subfamily B, polypeptide 7 pseudogene 1 | 2.3 | 0.009 | −2.3 | 0.01 |
| 15360 | PLTP | phospholipid transfer protein | 10.2 | 0.0091 | −3.3 | 0.02 |
| 94009 | SERHL | serine hydrolase-like | 5.6 | 0.0097 | −2 | 0.047 |
| 84873 | GPR128 | G protein-coupled receptor 128 | 2.7 | 0.0098 | −3.4 | 0.006 |
| 374407 | DNAJB13 | DnaJ (Hsp40) homolog, subfamily B, member 13 | 3.7 | 0.0099 | −2.2 | 0.028 |
| 183 | AGT | angiotensinogen (serpin peptidase inhibitor, clade A, member 8) | 4.6 | 0.01 | −3.5 | 0.01 |
| 29943 | PADI1 | peptidyl arginine deiminase, type I | 2.8 | 0.01 | −2.3 | 0.02 |
| 2172 | FABP6 | fatty acid binding protein 6, ileal | 2 | 0.01 | −2 | 0.02 |
| 3249 | HPN | hepsin | 4.3 | 0.01 | −4.6 | 0.009 |
| 55018 | C17orf73 | chromosome 17 open reading frame 73 | 4.4 | 0.01 | −2.7 | 0.04 |
| 347 | APOD | apolipoprotein D | 4.9 | 0.01 | −2.3 | 0.04 |
| 80740 | LY6G6C | lymphocyte antigen 6 complex, locus G6C | 4.3 | 0.01 | −2.4 | 0.03 |
| 203328 | SUSD3 | sushi domain containing 3 | 5.2 | 0.01 | −2.5 | 0.03 |
| 3718 | JAK3 | Janus kinase 3 | 2.5 | 0.01 | −2.9 | 0.01 |
| 8514 | KCNAB2 | potassium voltage-gated channel, shaker-related subfamily, beta member 2 | 2.1 | 0.01 | −3.2 | 0.003 |

**Table S1.** *Cont.*

| Entrez gene ID | Gene symbol | Description | Upregulation (NaB *vs.* unstimulated) | | Downregulation (NaB + cip *vs.* unstimulated) | |
|---|---|---|---|---|---|---|
| | | | Fold change | *p*-value | Fold change | *p*-value |
| 7365 | UGT2B10 | UDP glucuronosyltransferase 2 family, polypeptide B10 | 2.9 | 0.01 | −2 | 0.04 |
| 2941 | GSTA4 | glutathione S-transferase alpha 4 | 2.3 | 0.01 | −4.2 | 0.002 |
| 7941 | PLA2G7 | phospholipase A2, group VII (platelet-activating factor acetylhydrolase, plasma) | 3.9 | 0.01 | −2.8 | 0.03 |
| 140738 | TMEM37 | transmembrane protein 37 | 3 | 0.017 | −4.2 | 0.01 |
| 118663 | BTBD16 | BTB (POZ) domain containing 16 | 2.1 | 0.017 | −3.9 | 0.004 |
| 7739 | ZNF185 | zinc finger protein 185 (LIM domain) | 2 | 0.017 | −2.4 | 0.01 |
| 64600 | PLA2G2F | phospholipase A2, group IIF | 12.4 | 0.018 | −4.2 | 0.03 |
| 64220 | STRA6 | stimulated by retinoic acid gene 6 homolog (mouse) | 6.3 | 0.018 | −5.9 | 0.02 |
| 5972 | REN | renin | 6.7 | 0.018 | −8.3 | 0.016 |
| 3827 | KNG1 | kininogen 1 | 6.1 | 0.018 | −3.8 | 0.02 |
| 25809 | TTLL1 | tubulin tyrosine ligase-like family, member 1 | 2 | 0.019 | −3.7 | 0.002 |
| 10232 | MSLN | mesothelin | 3 | 0.019 | −3.9 | 0.01 |
| 6947 | TCN1 | transcobalamin I (vitamin B12 binding protein, R binder family) | 7 | 0.019 | −3.4 | 0.04 |
| 3748 | KCNC3 | potassium voltage-gated channel, Shaw-related subfamily, member 3 | 2 | 0.019 | −2.1 | 0.01 |
| 9022 | CLIC3 | chloride intracellular channel 3 | 3.1 | 0.02 | −5.3 | 0.01 |
| 80896 | NPL | N-acetylneuraminate pyruvate lyase (dihydrodipicolinate synthase) | 3.1 | 0.02 | −2.1 | 0.04 |
| 79745 | KLIP4 | CAP-GLY domain containing linker protein family, member 4 | 2.8 | 0.02 | −2.4 | 0.03 |
| 8736 | MYOM1 | myomesin 1, 185 kDa | 2.5 | 0.02 | −2.5 | 0.02 |
| 3557 | IL1RN | interleukin 1 receptor antagonist | 3.4 | 0.02 | −3.2 | 0.04 |
| 4485 | MST1 | macrophage stimulating 1 (hepatocyte growth factor-like) | 2.4 | 0.02 | −2.4 | 0.02 |
| 339416 | ANKRD45 | ankyrin repeat domain 45 | 4.9 | 0.02 | −12.6 | 0.01 |
| 5175 | PECAM1 | platelet/endothelial cell adhesion molecule | 2.3 | 0.02 | −2.5 | 0.02 |
| 251 | ALPPL2 | alkaline phosphatase, placental-like 2 | 18.1 | 0.02 | −11.13 | 0.03 |

**Table S1.** *Cont.*

| Entrez gene ID | Gene symbol | Description | Upregulation (NaB *vs.* unstimulated) | | Downregulation (NaB + cip *vs.* unstimulated) | |
|---|---|---|---|---|---|---|
| | | | Fold change | *p*-value | Fold change | *p*-value |
| 128553 | TSHZ2 | teashirt zinc finger homeobox 2 | 6.2 | 0.03 | −8.45 | 0.02 |
| 6799 | SULT1A2 | sulfotransferase family, cytosolic, 1A, phenol-preferring, member 2 | 2.9 | 0.03 | −4.21 | 0.02 |
| 387590 | psiTPTE22 | TPTE pseudogene | 4.3 | 0.03 | −4.8 | 0.02 |
| 84058 | WDR54 | WD repeat domain 54 | 2 | 0.03 | −2.3 | 0.02 |
| 2517 | FUCA1 | fucosidase, alpha-L-1, tissue | 2 | 0.03 | −3.2 | 0.007 |
| 53841 | CDHR5 | cadherin-related family member 5 | 2.1 | 0.03 | −2.4 | 0.01 |
| 79812 | MMRN2 | multimerin 2 | 2.7 | 0.03 | −4.7 | 0.01 |
| 127534 | GJB4 | gap junction protein, beta 4, 30.3kDa | 2.3 | 0.03 | −2.5 | 0.02 |
| 79861 | TUBAL3 | tubulin, alpha-like 3 | 2.8 | 0.03 | −2.4 | 0.03 |
| 283537 | SLC46A3 | solute carrier family 46, member 3 | 3.1 | 0.03 | −2.3 | 0.04 |
| 143662 | MUC15 | mucin 15, cell surface associated | 17.2 | 0.03 | −4.7 | 0.05 |
| 2346 | FOLH1 | folate hydrolase (prostate-specific membrane antigen) 1 | 2.1 | 0.03 | −5.2 | 0.008 |
| 4680 | CEACAM6 | carcinoembryonic antigen-related cell adhesion molecule 6 (non-specific cross reacting antigen) | 3.6 | 0.04 | −3.7 | 0.04 |
| 10170 | DHRS9 | dehydrogenase/reductase (SDR family) member 9 | 3.3 | 0.04 | −3.6 | 0.03 |
| 8529 | CYP4F2 | cytochrome P450, family 4, subfamily F, polypeptide 2 | 7.8 | 0.04 | −41.1 | 0.03 |
| 729359 | PLIN4 | perilipin 4 | 2.7 | 0.04 | −2.4 | 0.03 |
| 11045 | UPK1A | uroplakin 1A | 2.2 | 0.04 | −2.1 | 0.03 |
| 722 | C4BPA | complement component 4 binding protein, alpha | 15.7 | 0.04 | −10.9 | 0.047 |
| 8302 | KLRC4 | killer cell lectin-like receptor subfamily C, member 4 | 3.2 | 0.04 | −4.2 | 0.02 |
| 6819 | SULT1C2 | sulfotransferase family, cytosolic, 1C, member 2 | 3.1 | 0.04 | −3.8 | 0.03 |
| 15267 | SERPINA4 | serpin peptidase inhibitor, clade A (alpha-1 antiproteinase, antitrypsin), member 4 | 2.2 | 0.04 | −6.3 | 0.008 |
| 2984 | GUCY2C | guanylate cyclase 2C (heat stable enterotoxin receptor) | 3.7 | 0.047 | −5.34 | 0.03 |
| 11182 | SLC2A6 | solute carrier family 2 (facilitated glucose transporter), member 6 | 2.1 | 0.047 | −3.8 | 0.009 |
| 9963 | SLC23A1 | solute carrier family 23 (nucleobase transporters), member 1 | 2.2 | 0.048 | −2.8 | 0.03 |
